# Socially Oriented Approaches To Working with Children of Parents with Severe and Enduring Mental Illness: Expert Perspectives

**DOI:** 10.1007/s10597-025-01470-z

**Published:** 2025-10-06

**Authors:** Imogen Nevard, Helen Brooks, Judith Gellatly, Fritz Handerer, Penny Bee

**Affiliations:** 1 Division of Nursing, Midwifery & Social Work, School of Health Sciences, Faculty of Biology, Medicine and Health, The University of Manchester, 333 Jean McFarlane Building, Oxford Road, Manchester, M13 9PL UK; 2 Division of Psychology and Mental Health, School of Health Sciences, Faculty of Biology, Medicine and Health, The University of Manchester, Manchester, UK; 3 Oxford Institute of Clinical Psychology Training and Research, Medical Sciences Division, University of Oxford, Oxford, UK

**Keywords:** Social Networks, Social Network Analysis, Parental Mental Illness, Children of Parents with Mental Illness, Children and Youth, Community Mental Health Services

## Abstract

Children Of Parents with severe and enduring Mental Illness (COPMI) face an elevated risk for inherited mental health issues and diminished quality of life across various domains. While social factors such as social networks (the set of active, valued social ties surrounding an individual) are recognised as protective, they are often inadequately conceptualised, preventing effective leverage to promote positive outcomes. This brief report provides information regarding common network related issues faced by families, opportunities for supportive intervention, barriers and facilitators to social network conscious work with COPMI according to professionals. Professionals who work with individuals or families affected by parental mental illness provided insights as to how social network considerations can or do feature in their work via focus group discussions. Focus group transcripts were analysed through an a priori framework developed through framework analysis in order to identify common issues, potential interventions, and barriers and facilitators in their work. Commonly observed issues within family networks included the impact of caring roles, structural limitations to networks, and experiences related to stigma and trust. Network related intervention opportunities included early identification, support for community integration efforts, and child skills building. Barriers included lack of needs identification, communication gaps, and staff workload pressures. Potential facilitators include ongoing training, interdisciplinary collaboration, and consistency in staff/family relationships. This brief report offers valuable insights for practitioners, policymakers, and researchers emphasising the utility of relational approaches when working with families affected by parental mental illness.

## Introduction

Children of parents with severe and enduring mental illness (COPMI) are at elevated risk for the inheritance of intergenerational mental health issues as well as other negative physical, educational, psychosocial and quality of life outcomes compared to other children (Breslend et al., 2019; Dreyer et al., 2018; Foster et al., 2005; Nicholson et al., 2008; Oyserman et al., 2000; van Santvoort et al., 2015). Access to social support or social capital is a commonly ascribed protective factor, but one that is rarely fully operationalised as a variable in research and practice despite relational thinking presenting a promising avenue for mental health research and service provision (Nevard et al., 2021; Bjørnskov & Sønderskov, 2013; Bhandari & Yasunobu, 2009; Boden-Stuart & Larkin, 2024). Approaches from the field of social network analysis provide a framework for conceptualising social factors more concretely when conceptualising research, prevention, intervention and service provision. This study utilises egocentric network approaches, defining a social network as the set of active, valued ties surrounding an individual, in this case the child with a parent affected by severe and enduring mental illness (Vassilev et al., 2014). This structure of ties surrounding the child can act as a vector of support, learned skills and norms, all of which present opportunities to enhance or improve quality of life for the child.

Social network ties have the potential to influence the individual’s capacity to manage issues and stressors, including parental mental health status and include both informal ties and professional connections. Network ties act as a vehicle for instrumental, informational, emotional and recreational support, although they can also at times be a source of interpersonal strain (Nevard et al., 2024a). Network embeddedness, the degree to which an individual is integrated in their wider network, is overall associated with positive outcomes for vulnerable children (Nevard et al., 2021; Woolcock, 1998). If professionals work in a social network conscious way, i.e. are conceptually aware of the role and function of social ties as a protective factor, they are more likely to be able to leverage network related to promote positive outcomes for the child. Interventions which define how, and which ways they promote social outcomes such as access to support and network embeddedness will be more theoretically coherent, and better able to measure impact in evaluation stages.

This study aimed to collate and generate intervention and implementation specific findings from professionals working within the social networks of COPMI, to inform strategies targeted toward optimising social networks to best support well-being and quality of life of COPMI. The objectives of this research phase were to gather feedback on the relevance of network models such as the COPMI-NEM for diverse professionals working with COPMI populations and to explore how social network conscious (i.e. conceptually aware of the role and function of social ties as a protective factor) practices could be integrated into their approaches to working. The COPMI-NEM is a model which highlights the dynamic, fluctuating and often invisible needs of COPMI. It explores how networks are often poor at identifying these needs, fail to adequately respond to them, particularly over time.

Collated focus group data were both inductively and deductively analysed data to identify key insights and understanding from professionals about the role, characteristics and function of social networks in supporting the well-being and resilience of COPMI. This brief report presents preliminary findings particularly from the initial stage of analysis. This application of social network theory to the COPMI demographic addresses a gap in the literature, as evidenced by systematic reviews (Nevard et al., 2021; Stiawa & Kilian, 2017).

## Methods

We report intervention and implementation specific findings from a research study collecting perspectives from a diverse group of professionals actively engaged in supporting COPMI. The study brought together expertise from various domains, including adult and child mental health professionals including clinical psychologists (six), psychotherapists (five), a mental health nurse and one occupational therapist; researchers from both academic (four) and third sector (one) settings including those developing and trialling interventions for families affected by Severe and Enduring Mental Illness (SMI), educational professionals including teachers (two), teaching assistants (one) and special needs coordinators (one), and third sector professionals working with families where a parent has SMI (two). Many brought perspectives from a range of job roles throughout their professional career, or were currently holding dual roles; as such the total roles represented exceeds the sample size.

Participants had accrued relevant professional experience working with SMI affected families in a range of settings including inpatient, community, primary and secondary mental health services, schools, charities and the criminal justice system as well as academic research, intervention development and implementation and service delivery. They were recruited purposively through direct contact and snowball sampling.

In stage one of this research, a total sample of 17 experts participated in online focus groups with two researchers presenting a preexisting network model (COPMI-NEM) describing children’s dynamic interactions between formal and informal networks (Nevard et al., 2024b). The objectives outlined in the introductory section informed the development of topic guides for focus groups.Topic guides consisted of prompts including description of a social network model and open invitation to provide perspectives or feedback on its relevance for their work, as well as general perspectives on network conscious working. Prompts included preliminary questions ascertaining if social networks are a familiar conceptual construct and direct questions about how much social network concerns feature in their assessment and interactions with families, such as whether they collect network data or consider network changes to be a useful or relevant metric. Finally a series of optional open questions were curated to prompt discussion as needed, including such questions as ‘what information about family social networks would you find useful in your work?’ and what barriers would you expect to face if attempting to integrate social network considerations into your practice?’.

Transcripts were subject to a framework analysis which involved applying an a priori framework and subsequently developing an inductive a posteriori framework; the results presented here come from the application of preformulated a priori framework to transcripts, specifically geared towards identifying issues, challenges, opportunities, barriers and facilitators to network conscious working(Gale et al., 2013). Predetermined themes were generated based on these categories and the data was deductively reviewed and coded for: positive or negative reflections on the model and general construct of social networks, network practices (such as network data collection in assessment or ongoing work or conduct of network meetings), conceptual/attitudinal positions (such as beliefs about children, families, mental illness or childhood) and ideas about network conscious work including: family issues identified current practice, intervention opportunities and recommendations, barriers and facilitators.

In stage two, these findings were subsequently presented to a national group of research experts drawn from university institutions who provided context by way of ranking themes in terms of relevance and importance using a modified delphi method (Furber, 2010; Pan et al., 1996; Bourrée et al., 2008; Keeney et al., 2006; Strasser, 2017). The modified delphi consisted of a one hour estimate-talk-estimate approach delivered in an online meeting of a pre existing research network group. Attendees live ranked issues and opportunities in order of importance, and rated barriers and facilitators on Likert scales using interactive online software. The content was discussed as a group and new rankings were submitted at the end of the meeting. 11 individuals contributed to the rankings.

This study adhered to ethical guidelines, obtaining informed consent from all participants in focus groups and ensuring the confidentiality of their data. Consent taking was not required at the national network meeting, as verified by the University of Manchester’s Ethical Review Manager ethical decision tool (University of Manchester, 2023). This is because participants were recruited based on professional expertise, rather than lived experience. The original focus groups were approved by the University of Manchester Proportionate Ethics Committee, written consent was taken from participants and is securely stored at the University of Manchester (University of Manchester, 2024. This research received no specific grant from any funding agency in the public, commercial, or not-for-profit sectors, but builds upon prior work funded by the National Society for the Prevention of Cruelty to Children.

## Results

Results shown present the identified themes from the analysis of focus group data and are broken into four a priori themes, then further divided into inductively gathered sub themes. The first theme is issues commonly identified by professionals working with families affected by parental mental illness, related to their networks.

### Network issues

The first theme collates several challenges existing within the networks of families affected by parental mental illness, as identified by professionals. These issues include both individual and systemic factors that impede effective support for these children. These are (i) **Caring roles preventing network engagement**. The child’s caregiving role for the parent limits their capacity for network interactions including recreation. (ii) **Lack of parental network**. Many parents themselves lack a network of support, making it difficult for them to provide necessary assistance for their children. (iii) **Loss of network ties over time**. Both formal and informal supportive ties diminish over time, reducing overall support available for the family.

(iv) **Lack of parental willingness to engage with services**. Professionals reported that parents are reluctant to engage with formal networks due to fears that children may be removed from their care. This limits identification of COPMI and makes service provision less effective and accessible. (v) **Lack of parental willingness to engage with social ties**. Similarly, the impact of social stigma makes parents wary of seeking support within informal networks. (vi) **School transitions**. Transitioning between schools significantly disrupts children’s networks, making it harder for them to consistently access support.

(vii) **Lack of child willingness to engage with services.** Children are often wary of formal networks, due to their parents’ fears of child removal from the home, or from previous negative experiences with services leading to reduced uptake. (viii) **Lack of child willingness to engage with social ties.** As with their parents, children affected by social stigma may be less willing to engage with social connections, although this is considered a less significant phenomenon compared to parental reluctance.

These network issues are those which professionals working with families affected by parental SMI most commonly report observing in their practice. These issues prevent parents and children from building effective support networks, due to inconsistency of network ties, both formal and informal, and the impact of social stigma and care burdens. Disruptions, including changes such as school transition limit access to functional support for these families.

### Network Intervention Opportunities

Professionals from mental health services and from education also identified a number of opportunities for intervention on network levels, to improve outcomes for COPMI. These include systemic changes and proactive intervention strategies designed to strengthen networks and provide targeted support. These are (i) **Identification of COPMI**. Early recognition of children as affected by parental mental illness is critical for the facilitation of preventive measures, before the onset of network deterioration and other negative outcomes associated with COPMI status. (ii) **Identification of parental SMI**. Similarly, parental diagnosis identification is a key step which can help toward child identification.

(iii) **Parenting support**. Parenting interventions which acknowledge relational factors, including psychoeducation, guidance to emotionally support children, and assistance in building and maintaining social networks. (iv) **Community integration**. Families affected by SMI benefit from assistance in integration into broader community networks, including school communities, to generate sustainable support systems. Integration measures need to be sensitive to social stigma related to SMI. (v) **Clear information about available support**. Transparent, highly accessible information about support services is a crucial part of encouraging help-seeking behaviours to help families navigate formal networks. Professionals within formal networks will also benefit from up to date information on support available for these families.

(vi) **Relational skills building**. Teaching children and parents how to navigate social networks, improve communication skills, manage stigma in social interactions and to foster healthy relationships is key to building support systems. (vii) **Positive messaging regarding help seeking**. Within network messaging that promotes the acceptability of help seeking, whether in schools, or mental health services are required to encourage families to both seek and accept support. (viii) **Improving school attendance**. School attendance is key for both identification of support needs, and access to assistance. Non-attendance and school exclusion prevent help-seeking and access to formal networks. (ix) **Promoting network diversity**. Although a lower priority, diverse and inclusive networks can help children to access a wider range of information, perspectives and support types, including whistle blowing for abusive situations.

These intervention opportunities identified by professionals emphasise the need for early identification of families, preventative, relational support including skills building, and community integration to generate strong and diverse support networks for COPMI. Strengthening networks, by tackling stigma and providing targeted support may enhance access to resources through networks and improve quality of life.

### Barriers to Network Conscious Work

The 17 professionals identified a number of barriers to network conscious working in their profession. These highlight both systemic issues within formal systems and family level dynamics that limit network intervention efforts. Barriers to network oriented working are (i) **Poor identification of COPMI**. Children are not systematically identified as being affected by parental mental illness in formal systems, preventing timely intervention before negative impacts are realised. (ii) **Lack of communication between professionals**. A lack of communication between different members of families formal networks impedes effective collaboration and coordination of care. This is caused by technical, systemic and attitudinal issues.

(iii) **Staff workload**. High workloads and time pressures faced by professionals prevent them from allocating sufficient attention and resources to COPMI, particularly when their primary role is related to other areas such as the parent or educational outcomes. (iv) **Identification of dynamic parental support needs**. Parental SMI is chronic and episodic. It can be challenging for professionals to be aware and responsive to changing needs related to mental health states. This can lead to families receiving support from formal networks in crisis, when challenges become overtly visible to network members. (v) **Rigid codes of practice**. Strict adherence to formal codes of practice prevent more holistic and personalised support to families.

(vi) **Parental willingness to engage.** Parents can be reluctant to engage with formal networks due to fear of losing custody of their children, preventing professionals from establishing ties with families. (vii) **Budget allocation away from support roles.** Professionals report the diversion of resources away from pastoral or support roles geared towards identification of children’s support needs, particularly in schools. (viii) **Parental capacity**. Parental symptomatology can prevent parents from being able to support children’s engagement with networks, e.g. attending groups or activities.

(ix) **Performance focus over relational approaches**. A focus on performance metric such academic outcomes, rather than building relationships, can limit capacity for professionals to provide support. (x) **Perceptions of children’s role**. A miscomprehension of a child’s role and capacity can cause professionals to overlook opportunities to include the child, particularly when working with the parent’s mental health. (xi) **Access barriers**. Practical challenges, such as transport and logistics prevent children from engaging with their communities.

(xii) **Lack of advocacy.** There are a lack of advocacy roles to support families affected by parental mental illness in the navigation of complex formal networks. (xiii) **Child willingness**. Stigma surrounding parental SMI can prevent children from engaging with formal networks, although this was considered a low level barrier. (xiv) **Ancillary staff**. Again, a low level barrier, administrative staff are often gatekeepers for service access; where there is a lack of understanding of parental SMI on their part, families may be overlooked by formal networks.

Barriers then are primarily systematic, consisting of communication gaps, rigid practices, a lack of training and limited resources. The symptomatology of SMI, combined with family reluctance to engage with services due to stigma further hamper successful COPMI identification and intervention. This, paired with practical challenges and logistical barriers, can prevent professionals from successfully supporting families.

### Facilitators of network Conscious work

Professionals also identified factors that would enhance their ability to effectively provide network conscious support for these families. These facilitators prioritise systemic change, including training, communication and increased resources. Facilitators are (i) **Core staff training**. Ongoing, high quality training regarding the impact and significance of parental SMI is deemed crucial to improve network focused intervention. (ii) **Interdisciplinary communication and service integration**. Communication and collaboration across different disciplines including health and education will bridge gaps and help families to receive coordinated care. (iii) **Consistency in Relationships**. Maintaining consistent relationships between staff and families promotes trust which is vital for overcoming familial reluctance due to fear and stigma, for example by reducing staff turnover.

(iv) **Access to specialists and support staff**. When complex family needs exceed the capacity of professionals, they value reliable access to specialists to refer to, such as educational psychologists.(v) **Preventative mental health care**. Proactive mental health care that supports families before the point of crisis is considered essential to mitigate the most severe negative outcomes and promote integrated support. (vi) **Accessible information about service provision.** Both families and professionals require up to date and accessible information about available services. This helps professionals to signpost and for families to seek support, keeping formal networks integrated. (vii) **Clear**,** fully disseminated organisational policies.** Well-defined and widely shared organisational policies enable professionals to confidently and consistently offer appropriate support across sectors.

(viii) **Targets prioritising wellbeing over performance**. Work performance targets that allow professionals to target wellbeing in families, rather than purely outcome metrics such as academic performance encourages relational approaches when working with COPMI. (ix) **Training for ancillary staff**. Comprehensive staff training, including administrative and support staff can ensure that everyone engaging with families is appropriately equipped. (x) **Flexible codes of practice.** Adaptable frameworks, such as Open Dialogue which facilitate more flexible and creative operating procedures, while still upholding essential ethical commitments, can support interdisciplinary collaboration, as well as open communication between all members of formal and informal networks, better tailored to the personal needs of the family (Freeman et al., 2019; Galbusera & Kyselo, 2018).

(xi) **Accessible activities for children**. Affordable activities for children promotes community integration essential for network building. (xii) **Advocacy Roles**. Advocates can help integrate formal networks and assist families affected by parental SMI to navigate complex services and access support. (xiii) **Accessible transport.** Community integration efforts are only effective insofar as they are logistically viable. Limiting these barriers can improve family participation in local communities. (xiv) **Community events**. Community events, especially those within schools, provide opportunities for families to strengthen their networks and engage with both formal and informal networks to create supportive ties.

Training, flexible systems that prioritise communication and relational working, interdisciplinary collaboration, and accessible information about formal services available are all important facilitators when fostering a network conscious professional context. Additionally, maintaining consistent relationships between staff and family, and the provision of accessible transportation and community events are described as useful for strengthening networks. Professionals describe these factors as facilitative of their enhanced capacity to provide coordinated support for families that approaches them contextually within their social worlds.

## Discussion

This brief report explores the potentialities of network conscious working for professionals working with COPMI in clinical, educational, statutory and non statutory settings. It identifies key opportunities for application of network approaches and for network related intervention with these families, with a particular focus on the systematic changes required to enable effective professional practice. Relational approaches offer extensive potential for improving mental health service provision, particularly by utilising phenomenological concepts such as distress (Boden-Stuart & Larkin, 2024). A network focused approach that considers the child as inextricably part of their social context, offers professionals the opportunity to support the child in network navigation through and integration into networks. These factors are associated with positive outcomes that mitigate the impact of parental SMI on children; support and connectedness are both protective factors for COPMI (van Schoors et al., 2023; Nevard et al., 2024a).

COPMI are limited in their network engagement due to tangible factors such as caring load and intangible factors such as stigma related fears. Social ties may be limited in availability, quality or diversity. Individual professionals and structural systems can both assist in early identification of the parental illness and the affected child, and can facilitate community integration for the family. Professionals may encounter difficulties doing so due to a lack of appropriate information about the families, communication gaps between involved services and overall workload pressures. Participants in this study expressed a need for ongoing training, mechanisms for interdisciplinary collaboration and working structures that allow for consistency in relationships between affected families and staff. These elements were perceived to enhance their ability to view the child in a holistic way as a part of a wider network, and to support the child in their navigation of this network to promote wellbeing.

Structural approaches to work with vulnerable populations such as the application of ego network analysis and network models such as the COPMI-NEM (a model which illustrates the typical pattern of formal and informal network ties surrounding a child with a parent affected by SMI) offer utilisable explanatory frameworks by which professionals can understand how the child exists within a dynamic network of formal and informal ties (Nevard, 2024b). These frameworks can guide professionals in identifying and addressing network considerations such as the potential loss of ties over time, stigma related fears or the impact of their caring role on network engagement. Professionals can help children to leverage specific factors to enhance network embeddedness, identify supportive ties and navigate these ties in order to access support and improve their quality of life. Evidence from other populations demonstrates successful such interventions; a review found that intervention strategies for people with mental health problems should focus on supporting engagement with facilitated social activities outside of the mental health treatment context (Brooks et al., 2023). Professionals can also preempt social tie loss, promoting adaptive network navigation strategies in the face of stigma and fear, to prevent permanent erosion of children’s networks.

While social considerations and specific needs of parental SMI affected families are not novel concepts individually, this study offers a novel conceptual approach to integrate both perspectives, including a structural network approach rather than using generic terms such as support or connectedness to explore how professionals can use network conscious approaches to support COPMI. Specific, structural, network related observations, such as the loss of ties over time, the importance of school transitions as a particular period of loss, and the fluctuating needs of children based on fluctuating parental symptomatology and resultant need for care, highlights the importance of flexible, relationally driven and stigma aware approaches to support and intervention, in a way that more generic conceptions may not. This is a strength of this study, which provides a clear framework to for professionals conceptualise how formal and informal networks may fail to generate adequate support, and can hold in mind factors which may offset this. Factors identified by participants include promoting network diversity, assisting community integration and helping children in building relational, network navigation skills.

While a strength of the study is the variation in job roles represented, spanning a range of sectors and setting, a primary limitation is the relatively small sample size which may not represent perspectives of all relevant professionals. Despite recruitment efforts, social workers were not represented in focus groups, which posed a challenge to the study and created a gap in the dataset. While many social workers demonstrated initial interest, high work loads hindered involvement, and extension of data collection was not feasible within the project’s time constraints.

### Directions for research, policy and practice

The four themes explored in this study generate clear directions for institutional policies surrounding working with families affected by parental SMI. Workplaces can foster working practices that enhance the ability of their employees to enhance and promote supportive relationships with and between parents, children and services. Interdisciplinary working in this way can strengthen the ability of community mental health services to approach families in a holistic and relational manner, targeting networks before negative effects have had significant impacts upon children. Participants in these focus groups suggested drawing on flexible approaches, such as Open Dialogue to furnish new codes of practice which take adaptable, network oriented approaches to guidelines for standard practice which continue to meet professional ethical commitments. Operating procedures which facilitate consistent, ongoing relationships between professionals and families are likely to promote network conscious work and will be more responsive to fluctuating changes related to the episodic nature of chronic mental illness and its resultant impact on COPMI.

While social support and relational approaches were commonly used terminology by professionals and within workplaces, there was often a lack of clarity in the usage of these terms preventing the clear operationalisation of these relational factors as intervention points. This ambiguity prevents the implementation of meaningful targeted change for designated populations such as COPMI. Applying clearly defined network terminology and concepts to policy frameworks and practice guidance can help professionals to conceptualise and implement the holistic, relational working they are often guided towards, but do not always know how to action in practice (Cooklin et al., 2013). Using social network approaches to inform the development of policy and practice can facilitate community services to support meaningful network changes for service users, to foster positive relationships and improve personal support communities.

A shared language and understanding of network and relational concepts throughout professional workforces supporting COPMI are required to address systemic issues and achieve effective interdisciplinary working. Contextualising children as necessarily a part of their social worlds, and providing training as to what these social worlds may look like, professionals may be better placed to help families leverage social support from both formal and informal sources. This requires a move beyond superficial understandings of social relationships as important to service users, toward a detailed exploration of the structural and functional features of the social ties available to a family. This could include the accessibility, quality and diversity of these potential supportive relationships.

Taking this approach can transform abstract social concepts into practical tools. Professionals will be better placed to make sense of the social structures surrounding COPMI, personal communities composed of family members, friends, mental health and educational professionals, situated in community settings such as schools, wider families, social groups and cultural communities. These approaches should acknowledge that networks are dynamic vehicles of support for these children and their families. Social support and capital are often ideas at the centre of intervention efforts, however these are often approached superficially where social capital is constructed as the static, passive availability of assistance, which is conceptually problematic (Bjørnskov & Sønderskov, 2013; Bhandari & Yasunobu, 2009; Boden-Stuart & Larkin, 2024).

Network conscious approaches should consider how and in what ways an individual child can actively navigate social support including instrumental, emotional, informational and recreational from specific in network sources. This perceptual shift on the nature of supportive agents empowers children to strategically address their needs in network contexts. Interventions which actively foster skills and strategies for network navigation situate children as epistemic agents navigating their own networks and social worlds, actively participating in the creation of their own social support systems (Yates et al., 2024; Kirk, 2007).

Examples of network based intervention points include dissemination of ideas and messaging within networks to promote the acceptability of help seeking, the provision of accessible information about formal support and skills building groups or workshops to enhance network navigation strategies. These could focus on communication or relational skills, strategies for addressing and managing the effects of stigma and fostering help seeking behaviours. Approaches that actively equip children with the tools needed to effectively leverage and build upon pre existing networks appropriately situate COPMI as active agents. Centralising social networks in development of policy frameworks and intervention design and evaluation allows professionals to address the complex needs of COPMI, recognising the vulnerabilities and protective factors existing in their social worlds.

## Data Availability

The datasets presented in this article are not readily available because raw interview data is not provided publicly on the basis of confidentiality of participants.
